# Responses of Zooplankton Community Pattern to Environmental Factors along the Salinity Gradient in a Seagoing River in Tianjin, China

**DOI:** 10.3390/microorganisms11071638

**Published:** 2023-06-23

**Authors:** Xuewei Sun, Huayong Zhang, Zhongyu Wang, Tousheng Huang, Wang Tian, Hai Huang

**Affiliations:** Research Center for Engineering Ecology and Nonlinear Science, North China Electric Power University, Beijing 102206, China; xuewei_sun@ncepu.edu.cn (X.S.); zhy_wang@ncepu.edu.cn (Z.W.); 50902253@ncepu.edu.cn (T.H.); tianwang@ncepu.edu.cn (W.T.); huanghai@ncepu.edu.cn (H.H.)

**Keywords:** zooplankton community pattern, environmental factor, seagoing river, salinity, nutrient

## Abstract

As the primary consumers in aquatic organisms, zooplankton play an important role in aquatic ecosystems. It is valuable for management and researchers to have an insight into the responses of zooplankton community patterns to environmental factors. In this study, RDA and variation partitioning analysis were adopted to determine the important environmental factors affecting zooplankton abundance and biomass, as well as the relative importance of different environmental factors. The findings reveal that TN (total nitrogen), WD (water depth), pH, and SAL (salinity) were all important abiotic factors shaping the zooplankton community pattern in the study area. TN affected protozoa by influencing *Stentor amethystinus*, while the effects of WD on copepods may have been mainly induced by the responses of *Calanus sinicus* and *Paracyclopina nana*. By inhibiting *Stentor amethystinus* and *Vorticella lutea*, pH significantly affected protozoa. In addition, Rotifera and copepods were affected by SAL mainly through the responses of *Brachionus calyciflorus*, *Calanus sinicus*, and *Ectocyclops phaleratus*. Importantly, fundamental alternations in the variation trends of zooplankton abundance and biomass along the salinity gradient were found when the salinity was approximately 4–5. By combining these results with the findings on phytoplankton responses to salinity in previous studies, it can be concluded that salinity may influence the river ecosystem by influencing zooplankton abundance and biomass rather than phytoplankton.

## 1. Introduction

As the primary consumers in aquatic food chains, zooplankton feed on bacteria, debris, and phytoplankton, which can change the community structure of phytoplankton and further affect the function of the aquatic ecosystem through the trophic relationship of phytoplankton–zooplankton–fish [1]. Thus, zooplankton occupy an important position in aquatic ecosystems and play indispensable parts in maintaining biological balance [2]. In the assessment of water quality and ecosystem health, some zooplankton that are sensitive to environmental changes have often been selected as indicator species [3]. Therefore, exploring the relationship between the zooplankton community and water environmental factors is of great significance not only for aquatic ecosystem health and water quality monitoring and maintenance but also for further understanding the ecological processes in river ecosystems.

Zooplankton play important parts in transforming energy from lower-trophic organisms to higher ones, regulating phytoplankton growth, and shaping planktonic ecosystems [2,4]. Numerous previous studies on zooplankton have paid great attention to the effects of physicochemical environmental factors and nutrients on community parameters [5,6,7,8,9,10]. Local environmental factors, such as water temperature, pH, salinity, trophic state, or combinations of these factors, are responsible for shaping the local community structure (i.e., the species-sorting hypothesis) [11,12]. Moreover, nutrients are major environmental factors affecting zooplankton [5,10]. Nutrients can also affect zooplankton indirectly by affecting phytoplankton through a top-down effect.

In recent years, the effects of salinity on zooplankton have received much attention. Previous studies have revealed that salinity is an important environmental filter that may control the species composition and biomass of zooplankton communities in coastal estuarine ecology [7,13,14,15]. The direct or first-order effects of salinity result from changes in organisms’ osmoregulatory ability, food digestibility, and hormonal stimulation. In most cases, indirect effects are related to salinity-induced changes in competition patterns or the predation regime, which may be more important than direct salinity effects in explaining population dynamics and community structures in brackish water [5,6]. Therefore, it is better to select rivers with a significant salinity gradient to analyze the real interaction relationship. However, previous studies have only shown that salinity has an effect on zooplankton, but the importance of salinity relative to nutrients and other environmental factors remains unknown.

The Duliujian River is the largest river in the southern region of the Haihe River plain with a significant salinity gradient. Thus, it was selected as the research object to study the response of the zooplankton community to environmental factors. In this study, 18 abiotic factors and zooplankton data from 15 stations were monitored and analyzed in the river estuary ecosystem of the Duliujian River. The objectives of this study were to (i) identify the main factors affecting the zooplankton community’s abundance and biomass; (ii) quantify the relative importance of salinity impacts on the zooplankton community; and (iii) find potential thresholds for the effects of salinity on zooplankton community abundance and biomass.

## 2. Materials and Methods

### 2.1. Study Area

The Duliujian River in Tianjin City, China (39°3′20″–38°46′4″ N, 116°55′10″–117°33′44″ E) is the largest river in the southern Haihe River Basin’s lower portions. It flows from the confluence of the Daqing River and the Ziya River to Bohai Bay (Figure S1), with a total length of 70.14 km, a maximum width of 1 km in the upper and middle reaches, and a catchment area of 3737 km^2^. The river basin is located in the warm temperate zone and has a semi-humid continental monsoon climate, which means a unique seasonal climate. The annual average temperature ranges from 12 °C to 15 °C within the region, while the precipitation is concentrated from June to September, with an average annual rainfall of approximately 571 mm. Additionally, the Duliujian River connects two important reservoirs, the Tuanbowa Reservoir and the Beida Harbor Reservoir. Despite sluice control, interactions can possibly occur through small tributaries. Frequent human activities have caused point and non-point source pollution in the Duliujian River, such as factory wastewater discharge, urban domestic sewage discharge, and fishery and agricultural pollution, which could also increase the salinity in the river to a certain extent. The river’s proximity to the ocean renders it susceptible to saltwater intrusion. Consequently, the entire river exhibits a salinity gradient that, in conjunction with other abiotic conditions, influences the survival, growth, and reproduction of aquatic creatures [10]. Please refer to [10] for the specific study area and sampling station, and the map is depicted in the Supplementary Materials for the convenience of reviewers and readers.

### 2.2. Field Sampling and Laboratory Analyses

Water and zooplankton samples were collected quarterly from 15 stations along the studied river during the period from September 2017 to June 2018, obtaining a total of 45 water samples and 45 zooplankton samples per quarter. At each sampling location, three parallel water samples were taken with a polymethylmethacrylate sampler and stored in polyethylene plastic bottles. The water samples were then placed in insulated cartons and delivered within eight hours to the lab for additional analysis. The samples were handled and preserved in accordance with all applicable national industry norms and regulations. Water temperature (WT), water depth (WD), dissolved oxygen (DO), salinity (SAL), pH, and oxidation–reduction potential (ORP) were some of the environmental factors that were measured in situ (YSI ProPlus), while the other factors, such as total phosphorus (TP), total nitrogen (TN), total dissolved phosphorus (TDP), total dissolved nitrogen (TDN), ammonia–nitrogen (NH_4_^+^-N), nitrate–nitrogen (NO_3_^−^-N), nitrite–nitrogen (NO_2_^−^-N), and chemical oxygen demand (COD) were analyzed in the laboratory. The water samples were tested for TN, TP, COD, and TUR in the lab. The other samples, which had been filtered through 0.45 m filter membranes, were tested for the other nutrient factors. Every environmental factor was measured in accordance with the relevant standards (Table S1). Furthermore, the environmental factors had significant seasonal variations, and the distributions of important environmental factors are shown in the Supplementary Materials (Table S2 and Figure S2). For detailed descriptions of the environmental factors, please refer to the previous article [10], which was about the effects of environmental factors on the phytoplankton community. Since the sampling time of the zooplankton data in this paper was exactly the same as that of the phytoplankton data in the previous paper, the descriptions of the environmental factors are not described again in this paper in order to avoid duplication.

For copepods and Cladocera, 20 L water samples were repeatedly collected twice at a depth of 1 m and filtered with a 25# plankton collection net (with a mesh size of 0.064 mm), and the net was then washed once. Each filtrate was injected into a 50 mL polyethylene bottle and then fixed with a formaldehyde solution (4% *v/v*). After 24 h of standing, each supernatant fluid was slowly removed, and the final sample volume was 30 mL. At each station, three parallel samples were collected. For 3 parallel zooplankton samples of protozoa and rotifers, 1 L water samples were collected with polyethylene bottles at a 0.5 m surface water depth and immediately fixed with 10 mL of Lugol’s solution. In the laboratory, these samples were stored in glass containers for 24 h, and then the upper section of each solution was siphoned using a rubber hose. For each sample, the remaining 50 mL sample sediment was transferred into a polyethylene bottle and preserved by adding formaldehyde (4% *v/v*). After 24 h, it was condensed to 30 mL by sucking away the supernatant liquid with a pipette. Lastly, all the pretreated samples were well-packaged and transferred to the Center of Monitoring and Scientific Research of Ecology and Environment, the Administration of Ecology and Environment of Haihe Basin and Beihai Area, MEE, to identify zooplankton species and quantify zooplankton abundance and biomass.

### 2.3. Data Analysis

The dominance value (*Y*) of each species was calculated with Equation (1) to determine the dominant species of zooplankton.

(1) $$Y=\left(\frac{n_{i}}{N}\right)\times f_{i}$$

where $n_{i}$ is the individual number of species i within a given area, N is the total individual number of all species, and $f_{i}$ is the occurrence frequency of species i. Species were considered dominant during the sampling period if Y > 0.02.

In order to reduce sampling error, the data on environmental factors and zooplankton used for statistical analysis were the average values of three parallel samples of each sampling station in each quarter. Before statistical analysis, all species data and water environmental factors (except for pH) were log_10_(x+1)-transformed to eliminate dimensional differences. 

The responses of the zooplankton community abundance and biomass to environmental conditions were analyzed using redundancy analysis (RDA, vegan package, R, version 4.1.2). Prior to the RDA, detrended correspondence analysis (DCA) was used to assess whether linear or unimodal ordination methods should be implemented. In this study, all DCA values were less than 4, and RDA was, therefore, the most reliable model for analyzing the interactions between the zooplankton community and environmental conditions. The forward or backward selection approach with Monte Carlo permutation tests (999 permutations) was used to find the driving environmental determinants of the variance in the zooplankton community (function ordiR2step, adespatial package, R, version 4.1.2). Then, the variance inflation factor (VIF) was used to check the collinearity between the selected elements. The significance of the association between the zooplankton community and the filtered environmental parameters was evaluated using the Mantel test. Last but not least, the validity and significance of the RDA results were assessed using Monte Carlo permutation tests (999 permutations), and the results were significant when *p* < 0.05. In addition, Monte Carlo permutation tests (999 permutations) with Bonferroni adjustment were used to determine whether the first 2 constraint axes were relevant at a significance level of *p* < 0.05. Variance partitioning analysis (VPA) was employed to quantify the unique and shared fractions of variation in the zooplankton community, which were explained via SAL, NUT (environmental factors including nitrogen and phosphorous, e.g., TN and TDP), and PC (environmental factors except for SAL and NUT, e.g., pH, WD, and WT) (function varpart, vegan package, R, version 4.1.2). The zooplankton species names and corresponding codes can be checked in Table S3.

## 3. Results

### 3.1. Zooplankton Abundance and Relationships with Environmental Factors

**Figure 1:**
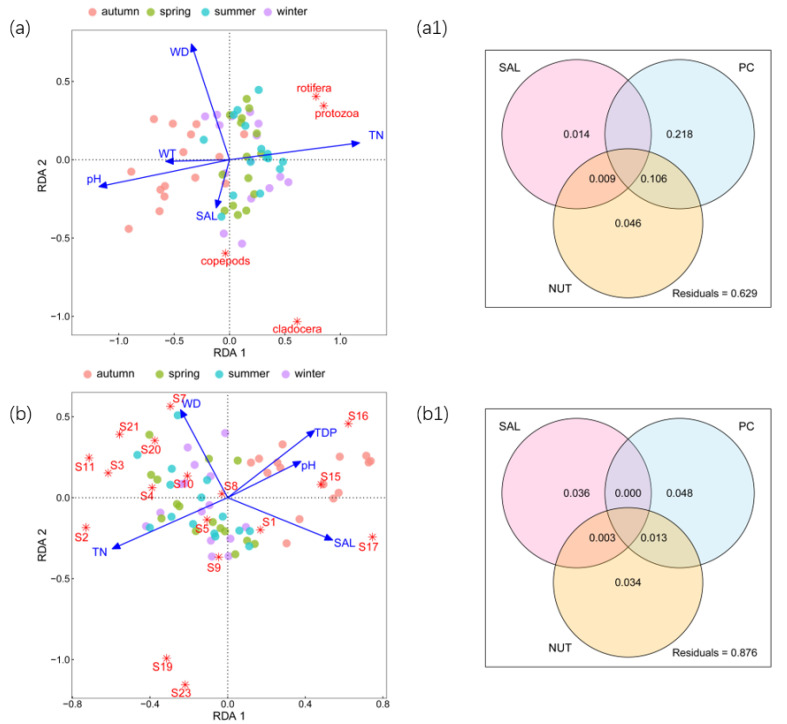
RDA and variation partitioning of zooplankton abundance. (**a**) RDA of zooplankton abundance at grouping level; (**a1**) results of variation partitioning of zooplankton abundance at grouping level; (**b**) RDA of zooplankton species abundance at species level; (**b1**) results of variation partitioning of zooplankton abundance at species level.

A total of 17 zooplankton species were identified in 4 seasons with abundance values ranging from 21.00 ind./L to 1218.33 ind./L, with an average of 471.48 ind./L (Figure S3). RDA was employed to analyze and present the responses of zooplankton abundance to environmental factors. For zooplankton abundance at the grouping level (Figure 1a), the RDA results were significantly confirmed via Mantel tests (r = 0.19; *p* < 0.01). Moreover, the first 2 constraint axes were meaningful at *p* < 0.001, which was proved using Monte Carlo permutation tests (999 permutations) with Bonferroni adjustment. It explained 36.38% of the total variance in the zooplankton abundance, of which the eigenvalue of axis 1 was 1.12, accounting for 28.16%, while the eigenvalue of axis 2 was 0.33, accounting for 8.22%.

As shown in Figure 1a, TN, WD, pH, SAL, and WT significantly affected zooplankton abundance at the grouping level. It was found that protozoa and cladocera had positive relationships with TN. WD had negative effects on copepods and cladocera, while protozoa and rotifera were negatively associated with pH. SAL was positively correlated with copepods and negatively correlated with rotifera. According to the results of the variation partitioning, the variation could be explained by PC unique fraction > NUT unique fraction > SAL unique fraction (Figure 1(a1)).

For zooplankton abundance at the species level (Figure 1b), the RDA results were significantly confirmed using Mantel tests (r = 0.17; *p* < 0.01). Moreover, the first 2 constraint axes were meaningful at *p* < 0.01, which was proved using Monte Carlo permutation tests (999 permutations) with Bonferroni adjustment. It explained 14.19% of the total variance in the zooplankton abundance, of which the eigenvalue of axis 1 was 1.48, accounting for 8.72%, and the eigenvalue of axis 2 was 0.93, accounting for 5.47%.

**Figure 2:**
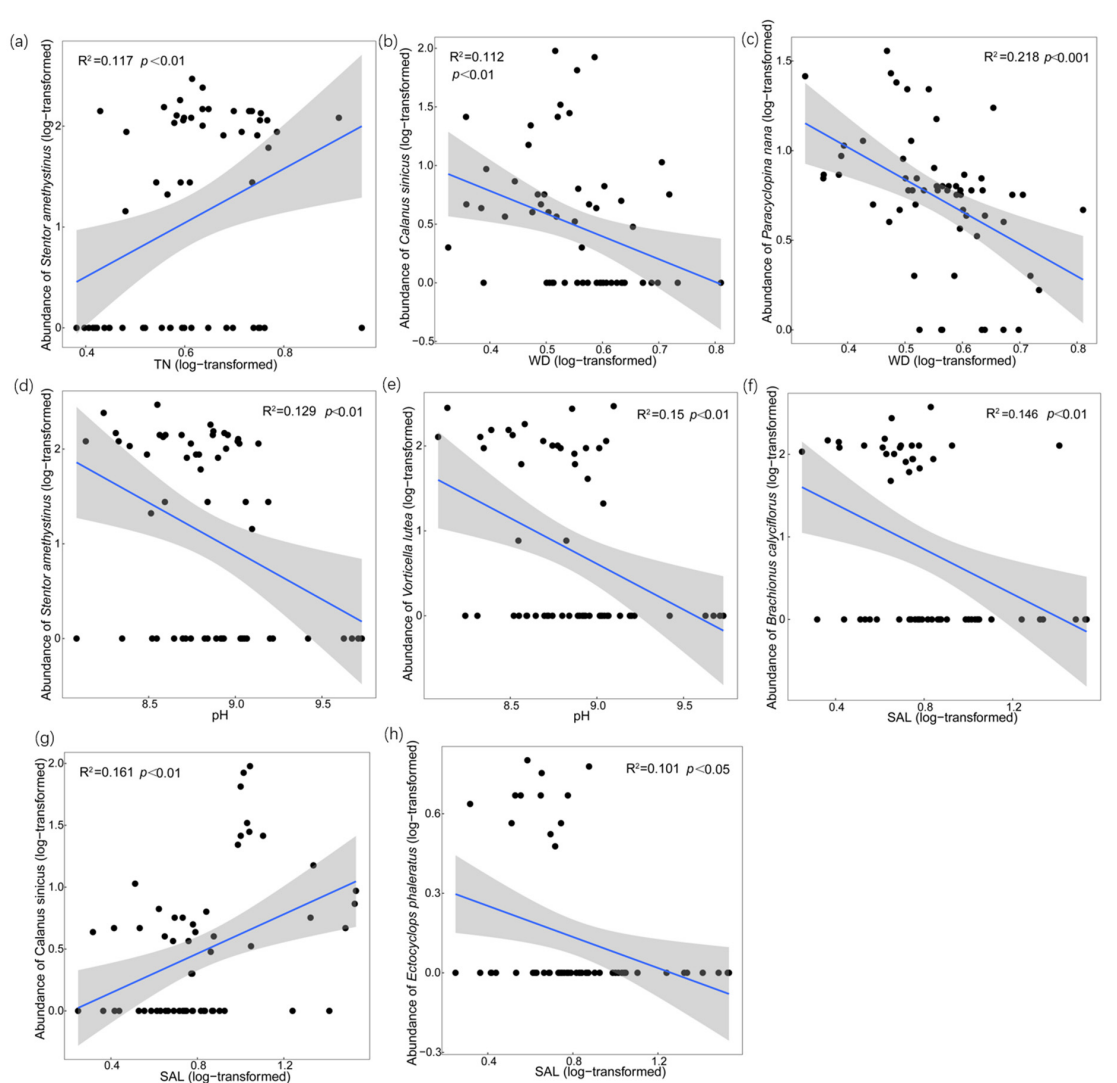
Influences of TN, WD, pH, and SAL on specific species’ abundance. (**a**) Linear regression of abundance of *Stentor amethystinus* and TN; (**b**) Linear regression of abundance of *Calanus sinicus* and WD; (**c**) Linear regression of abundance of *Paracyclopina nana* and WD; (**d**) Linear regression of abundance of *Stentor amethystinus* and pH; (**e**) Linear regression of abundance of *Vorticella lutea* and pH; (**f**) Linear regression of abundance of *Brachionus calyciflorus* and SAL; (**g**) Linear regression of abundance of *Calanus sinicus* and SAL; (**h**) Linear regression of abundance of *Ectocyclops phaleratus* and SAL. Black dots are abundance of zooplankton in different sampling station. Blue lines are the fitting lines of linear regressions. Grey shadow is the 95% confidence interval.

As shown in Figure 1b, the zooplankton abundance at the species level was mainly influenced by TN, WD, pH, SAL, and TDP. According to the results of the variation partitioning, the variation could be explained by PC unique fraction > SAL unique fraction > NUT unique fraction (Figure 1(b1)). It was found that *Stentor amethystinus* presented notable positive correlations with TN, and TN could explain 11.7% (*p* < 0.01) of the variation in *Stentor amethystinus* (Figure 2a). *Calanus sinicus*, *Paracyclopina nana*, and *Bosmina coregoni* were negatively correlated with WD, and WD could explain 11.2% (*p* < 0.01) and 21.8% (*p* < 0.001) of the variation in *Calanus sinicus* and *Paracyclopina nana*, respectively (Figure 2b,c). *Stentor amethystinus* and *Vorticella lutea* showed significant negative correlations with pH, and the influence could explain 12.9% (*p* < 0.01) and 15% (*p* < 0.01) of the variance, respectively (Figure 2d,e). In addition, there was a significant positive correlation between *Calanus sinicus* and SAL, with which 16.1% (*p* < 0.01) of the variation in *Calanus sinicus* could be explained (Figure 2g). *Brachionus calyciflorus* and *Ectocyclops phaleratus* had negative relationships with SAL, and the influence could explain 14.6% (*p* < 0.01) and 10.1% (*p* < 0.05) of the variation, respectively (Figure 2f,h).

### 3.2. Zooplankton Biomass and Relationships with Environmental Factors

**Figure 3:**
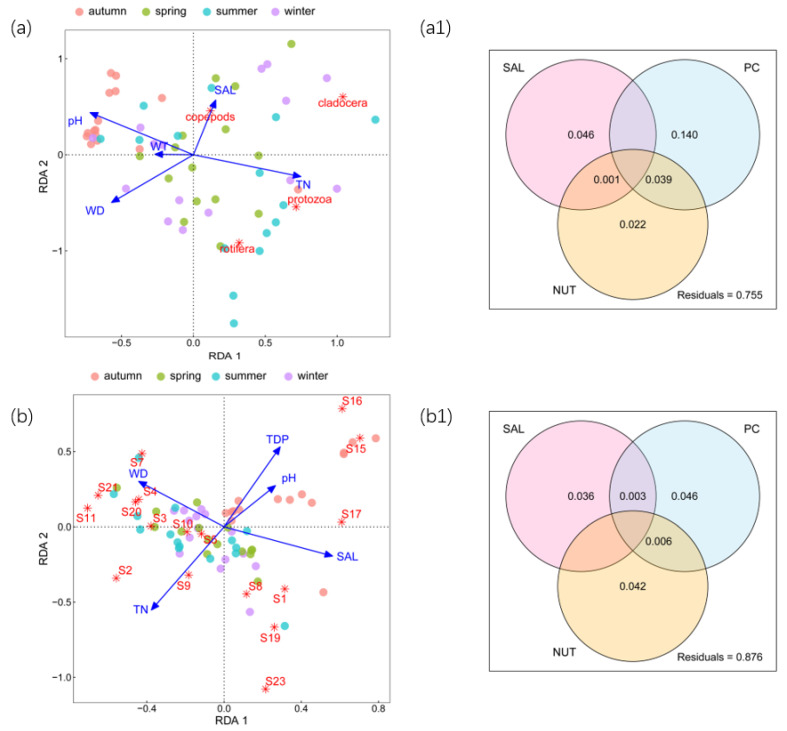
RDA and variation partitioning of zooplankton biomass. (**a**) RDA of zooplankton biomass at grouping level; (**a1**) results of variation partitioning of species biomass at grouping level; (**b**) RDA of zooplankton species biomass at species level; (**b1**) results of variation partitioning of species biomass at species level.

The biomass of zooplankton ranged from 0.004 mg/L to 18.17 mg/L, with an average of 4.08 mg/L (Figure S3). The RDA results for zooplankton biomass at the grouping level shown in Figure 3a were significantly confirmed using Mantel tests (r = 0.15; *p* < 0.01). Moreover, the first 2 constraint axes were meaningful at *p* < 0.01 and proved using Monte Carlo permutation tests (999 permutations) with Bonferroni adjustment, which explained 23.94% of the total variance in the zooplankton biomass. TN, WD, pH, SAL, and WT significantly affected zooplankton biomass at the grouping level. It was found that the effects of the environmental factors on zooplankton biomass were similar to those of the environmental factors on zooplankton abundance. Specifically, TN and pH had positive and negative correlations with both protozoa and rotifera, respectively, while WD was negative for copepods and cladocera. SAL had positive and negative correlations with copepods and rotifera, respectively. According to the results of the variation partitioning, the variation could be explained by PC unique fraction > SAL unique fraction > NUT unique fraction (Figure 3(a1)).

**Figure 4:**
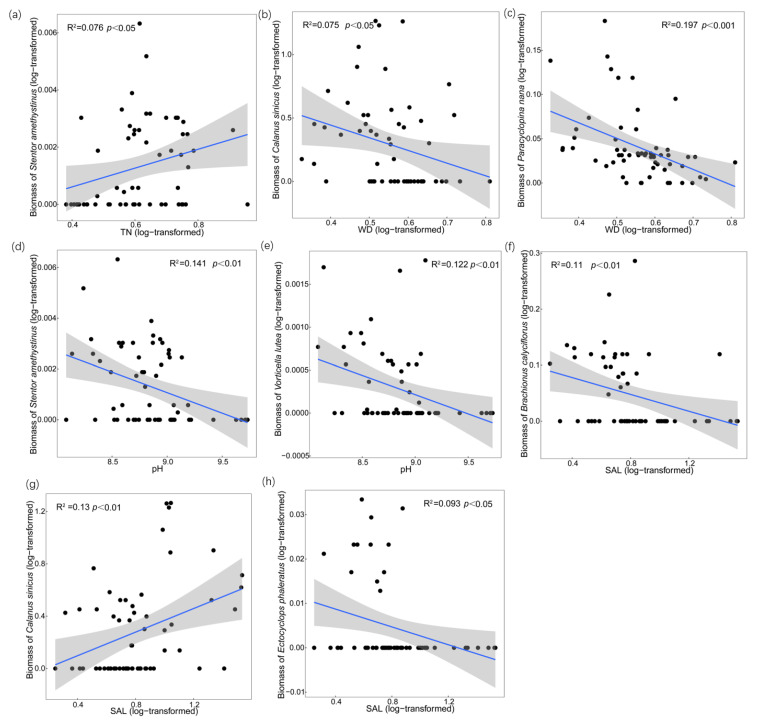
Influences of TN, WD, pH, and SAL on specific species’ biomass. (**a**) Linear regression of biomass of *Stentor amethystinus* and TN; (**b**) Linear regression of biomass of *Calanus sinicus* and WD; (**c**) Linear regression of biomass of *Paracyclopina nana* and WD; (**d**) Linear regression of biomass of *Stentor amethystinus* and pH; (**e**) Linear regression of biomass of *Vorticella lutea* and pH; (**f**) Linear regression of biomass of *Brachionus calyciflorus* and SAL; (**g**) Linear regression of biomass of *Calanus sinicus* and SAL; (**h**) Linear regression of biomass of *Ectocyclops phaleratus* and SAL. Black dots are biomass of zooplankton in different sampling station. Blue lines are the fitting lines of linear regressions. Grey shadow is the 95% confidence interval.

For the zooplankton biomass at the species level (Figure 3b), the RDA results were significantly confirmed using Mantel tests (r = 0.17; *p* < 0.01). The first 2 constraint axes were also meaningful at *p* < 0.01 and proved using Monte Carlo permutation tests (999 permutations) with Bonferroni adjustment. They explained 13.38% of the total variance in the zooplankton abundance, with the eigenvalues of axis 1 and axis 2 being 1.34 and 0.94 and accounting for 7.88% and 5.5%, respectively. As shown in the figure, zooplankton biomass at the species level was mainly influenced by TN, WD, pH, SAL, and TDP, which was similar to zooplankton abundance. According to the results of the variation partitioning, the variation could be explained by PC unique fraction > NUT unique fraction > SAL unique fraction (Figure 3(b1)). TN showed a positive relationship with *Stentor amethystinus*, and linear regression showed that TN could explain 7.6% (*p* < 0.01) of its variation (Figure 4a). *Calanus sinicus*, *Paracyclopina nana*, and *Bosmina coregoni* were negatively correlated with WD, which could explain 7.5% (*p* < 0.01) of the variation in *Calanus sinicus* and 19.7% (*p* < 0.001) of the variation in *Paracyclopina nana* (Figure 4b,c). *Stentor amethystinus* and *Vorticella lutea* showed significant negative correlations with pH, and 14.1% (*p* < 0.01) and 12.2% (*p* < 0.01) of their variations could be explained, respectively (Figure 4d,e). In addition, there was a significant positive correlation between *Calanus sinicus* and SAL, which could explain 13% (*p* < 0.01) of its dynamics (Figure 4g). *Brachionus calyciflorus* and *Ectocyclops phaleratus* had negative relationships with SAL, and 11% (*p* < 0.01) and 9.3% (*p* < 0.05) of their variations could be explained, respectively (Figure 4f,h).

## 4. Discussion

Li [8] studied the mesozooplankton community structure in Bohai Bay, and Yu investigated the plankton in the Tianjin section downstream of the Haihe River [16]. The zooplankton abundance and biomass in this study were consistent with previous studies [8,16]. For both abundance and biomass, TN, WD, pH, and SAL were all important environmental factors causing zooplankton changes at the community and population levels, but their relative importance was different. In addition, different groups of zooplankton had different responses to environmental factors.

TN was the driving factor of zooplankton community abundance and biomass changes and had different effects on protozoa, rotifera, and cladocera, which accounted for 92.01% of the total abundance of the zooplankton community. According to the regression relationships between species and environmental factors, there was a significant positive correlation between TN and *Stentor amethystinus*, which was the dominant species of protozoa, accounting for 37.14% of the abundance and 57.5% of the biomass of protozoa. Therefore, the effect of TN on protozoa may be realized mainly through its promoting effect on *Stentor amethystinus*. Studies have successfully identified the determinant role of TN on protozoa community structures. The TN values during the studied period, with an average concentration of 3.30 mg/L, indicated that there was high-level nutrient contamination in the studied area. The close positive relationship between *Stentor amethystinus* and TN that was observed must be attributed to both direct effects and indirect effects. The direct reason was that *Stentor amethystinus* had a relatively high tolerance for eutrophic status. Studies in fish culture ponds and Chaohu Lake have revealed that some protozoan species could even endure environmental conditions that were severe for macrofauna [17,18]. In addition, many protozoan species reacted quickly to pollutants and frequently lived in habitats that were adverse to most metazoans [17,18]. The indirect reason was the “top-down” effect. Previous studies have revealed that high nutrient concentrations can stimulate primary production, which facilitates the reproduction and growth of zooplankton and other predators that feed on primary producers [19]. In this study, the abundance and biomass of protozoa had significantly positive correlations with the biomass of Bacillariophyta and Cryptophyta (Table S4), which meant that TN provided a nitrogen source for phytoplankton, and thus indirectly provided a food source for protozoa and promoted their growth and development.

In previous studies, rotifera was reported to dominate total zooplankton within highly eutrophic waters [20]. However, the effect of nitrogen content on the rotifer community (positive or negative) has not been consistent. Xiong et al. identified the determinant role of total nitrogen on the rotifer community structure: the total abundance and species richness decreased with the increase in TN [12]. However, Wang et al. indicated that nitrogen could have a positive effect on rotifera [21]. As Xiong et al. have suggested, different results may be attributable to differences in the gradient range and/or gradient length of nitrogen concentration and the corresponding trophic states in the regions surveyed. In the study by Xiong et al., the range of TN concentrations was 0.6–30.0 mg/L, which represents a higher nutrient influx and a much longer environmental gradient length, whereas the range was 0.48–8.67 mg/L in Wang et al.’s study. In this study, similar results were obtained with a TN range from 1.41 to 8.07 mg/L, which was close to that of Wang et al. [21].

WD was an important environmental factor affecting zooplankton abundance and biomass, especially in copepods and cladocera. Consistent with many previous studies [22,23,24], the effects of WD on copepods may have been mainly induced by *Calanus sinicus* and *Paracyclopina nana*, which accounted for 93.41% of the biomass of copepods. The sampling depth was more important for the mesozooplankton community than the spatial distribution of the sampling stations, in spite of the large geographic extent of the study area [25]. The negative relationship between WD and copepods was consistent with a study in the tropical Southwestern Atlantic [26]. As a previous study reported, depth was likely one of the primary abiotic factors influencing the zooplankton distributions [27]. A higher water depth could present a complex hydraulic environment affecting food availability at this depth for *Calanus sinicus* [28] and *Paracyclopina nana*.

pH was the driving environmental factor of protozoa and rotifera at the community level, while at the species level, pH had significant negative effects on *Stentor amethystinus* and *Vorticella lutea*. In the studied area, pH affected protozoa primarily by inhibiting *Stentor amethystinus* and *Vorticella lutea*. Studies indicated that pH was the most significant factor controlling the protozoa [29,30]. Protozoa abundance decreased with an increase in pH, and a pH of 9.5 could promote the complete inhabitation of protozoa, while a pH between 8.5 and 9.5 could delay the growth of protozoa [30].

SAL was the main environmental factor affecting rotifera and copepods. The effects of SAL on rotifera and copepods may be induced by influencing *Brachionus calyciflorus*, *Calanus sinicus*, and *Ectocyclops phaleratus*, among which *Brachionus calyciflorus* accounted for 88.02% of the biomass of rotifera, and *Calanus sinicus* and *Ectocyclops phaleratus* accounted for 89.14% of the biomass of copepods. As a kind of freshwater strain, *Brachionus calyciflorus* showed a negative correlation with SAL, which was consistent with other studies [31,32]. *Calanus sinicus* is an important copepod in the shelf waters of the western North Pacific [33] and is usually adapted to a wide range of salinities [34]. Yu’s study indicated that the feeding rate, water filtration rate, and fecal discharge rate of *Calanus sinicus* increased initially and then decreased with an increase in salinity and peaked at 28 [35], which was similar to the trend between SAL and *Calanus sinicus* in this study.

Furthermore, in the RDA and variation partitioning results, we found that salinity played a significant role in the abundance and biomass of the zooplankton community.

As for zooplankton abundance, the effect of salinity at the levels of zooplankton species was even more pronounced than that at community levels due to the different scales of observation and study, which indicated that the promotion or inhibition effects of salinity on species in the same group were not consistent, and the relationship between zooplankton and salinity at different scales could reflect a different relationship.

Many studies have reported that salinity is an important factor affecting the zooplankton community. Ecologically, salinization or a rise in salinity alters zooplankton composition and abundance [14,15,36]. Gao et al. indicated that the variation in salinity in an estuary resulted in regional and seasonal alterations in the dominant species [37]. This is consistent with the fact that zooplankton composition obviously shifts in the studied area with salinity variations. Zakaria et al. and Spoljar et al. indicated that rotifera and cladocera had negative correlations with salinity, while copepods displayed a positive correlation with salinity [38,39]. In this study, cladocera and rotifera had negative correlations with salinity, while copepods had a significant positive correlation with salinity. In particular, *Calanus sinicus*, a dominant species of copepods, often occurred in the coastal waters and could be considered an indicator of the water environment [3]. 

**Figure 5:**
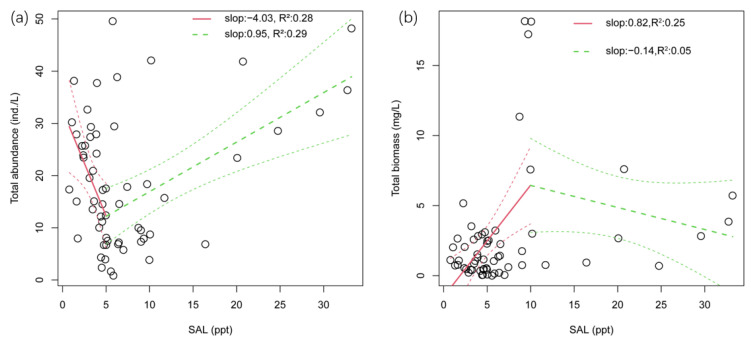
Effects of SAL on zooplankton total abundance and total biomass. (**a**) Segmented regression of total abundance and SAL; (**b**) Segmented regression of total biomass and SAL.

Previous studies have found that as salinity increases, zooplankton abundance and biomass decrease [14,15,36]. Consistent with previous studies, in this study, with the increase of salinity, species abundance showed a trend of decreasing first and then increasing, and the turning point was 5.02 ppt (*p* < 0.05) (Figure 5a), which was similar with the study in Hau River, Vietnam [7]. As for species biomass, it increased first and then decreased with the increasing salinity, and the turning point was 9.98 (*p* < 0.01) (Figure 5b). Changes in zooplankton abundance and biomass with salinity indicated that salinity affected zooplankton abundance and biomass, and regulated species composition. As salinity increases, zooplankton with a high salinity tolerance gradually weed out species with a low salinity tolerance and rapidly grow and reproduce. It has been proved via many studies that different concentrations of salinity have different effects on zooplankton [40,41,42], but herein, we tried to propose a relatively accurate salinity threshold. 

By analyzing the species distributions in freshwater and saltwater in the Baltic Sea, V. V. Khlebovich proposed the critical salinity concept, 5–8‰, in which the number of species reaches its minimum [43,44]. However, this is not a random occurrence, and this boundary is the result of a number of biological and abiotic changes in terms of organismic resistance to salinity, osmoregulation capacity, physiological activity, the electrochemical properties of tissues, and evolutionary aspects [43]. In this study, the turning point of zooplankton abundance was 5.02 ppt, and the turning point of zooplankton biomass was 9.98 ppt, which coincided to a great extent with the classical critical level (5–8 PSU). In this study, the abundance of zooplankton decreased initially and then increased as salinity increased, whereas the curve trend of biomass was the exact inverse, increasing initially and then decreasing as salinity increased. In this study, rotifera and protozoa with high abundance but low biomass were unable to adapt to an increasing-salinity environment and were replaced with copepods (such as *Calanus sinicus*) with low abundance but high biomass. In an investigation in the Neva Estuary, an opposite trend of rotifera and copepods with increasing salinity was also observed [45]. Therefore, the results of this research contribute, to some extent, to the study of critical salinity. In addition, combined with the effect of salinity on phytoplankton [10], salinity has no significant effect on phytoplankton abundance and biomass, but it has significant effects on zooplankton abundance and biomass. It can be inferred that salinity could regulate the river ecosystem by affecting zooplankton abundance and biomass.

## 5. Conclusions

In this study, RDA and variation partitioning analysis were used to determine the important environmental factors influencing zooplankton abundance and biomass, and the relative importance of different environmental factors. The results show that TN, WD, pH, and SAL were all crucial abiotic factors shaping the zooplankton community in this studied area. TN affected protozoa by affecting *Stentor amethystinus*. The effects of WD on copepods might have been mainly induced by *Calanus sinicus* and *Paracyclopina nana*. pH affected protozoa primarily by inhibiting *Stentor amethystinus* and *Vorticella lutea*. Rotifera and copepods were affected by SAL, which may have been induced by influencing *Brachionus calyciflorus*, *Calanus sinicus*, and *Ectocyclops phaleratus*. In addition, salinity of approximately 4–5 was important for the responses of the zooplankton community composition. Finally, combined with the results of previous studies on the effects of salinity on phytoplankton, it was found that salinity may adjust the river ecosystem by influencing the abundance and biomass of zooplankton rather than those of phytoplankton.

## Data Availability

The data can be made available upon request to the corresponding author.

## Supplementary Materials

The following supporting information can be downloaded at https://www.mdpi.com/article/10.3390/microorganisms11071638/s1: Figure S1: Location map of sampling station; Table S1: Analytical method for water environmental factors; Table S2: Environmental factors in Duliujian River. Max = maximum values, Min = minimum values, and SD = standard deviation; Figure S2: Seasonal variations in important environmental factors; Table S3: Zooplankton species names and corresponding codes. Figure S3. Seasonal variations in zooplankton abundance and biomass. (a) Seasonal variations in total abundance; (b) seasonal variations in total biomass. Table S4. Spearman’s correlation coefficients of biomass of Protozoa and biomass of Bacillari-ophyta and Cryptophyta. *** means *p* < 0.001, ** means *p* < 0.01, and * means *p* < 0.05. Ref. [46] is cited in the Supplementary Materials.
