# Supplementary material for: Responses of Zooplankton Community Pattern to Environmental Factors along the Salinity Gradient in a Seagoing River in Tianjin, China
Source: Microorganisms. 2023 Jun 23;11(7):1638. doi: 10.3390/microorganisms11071638 (PMC10384109; doi:10.3390/microorganisms11071638)
Supplement: Supplementary file 1 [file microorganisms-11-01638-s001.zip › microorganisms-2333778-supplementary.pdf]

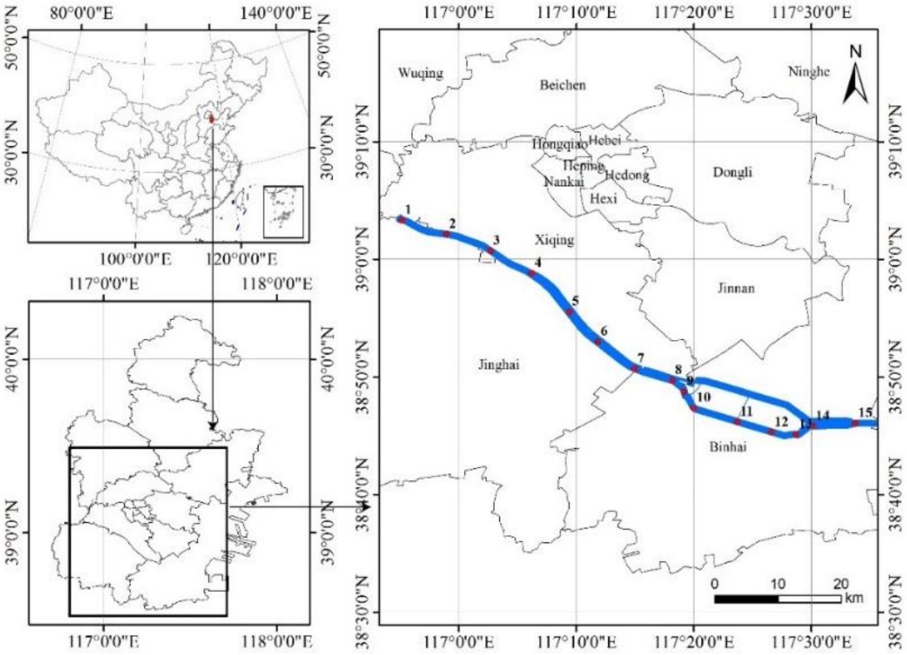

Figure S1. Location map of sampling stations.[1].

Table S1. Analytical method of water environmental factors [1,2].

Environmental factors	Abbreviation	Analytical method	Unit
Water temperature	WT	YSI ProPlus	°C
pH	pH	YSI ProPlus	
Dissolved oxygen	DO	YSI ProPlus	mg/L
Salinity	SAL	YSI ProPlus	ppt
Oxidation-reduction potential	ORP	YSI ProPlus	mv
Water depth	WD	Meter stick	m
Water transparency	SD	Secchi disk	cm
Total nitrogen	TN	Alkaline potassium persulfate digestion UV spectrophotometric method	mg/L
Total dissolved nitrogen	TDN	Alkaline potassium persulfate digestion UV spectrophotometric method	mg/L
Total phosphorus	TP	Ammonium molybdate spectrophotometric method	mg/L
Total dissolved phosphorus	TDP	Ammonium molybdate spectrophotometric method	mg/L
Chemical oxygen demand	COD	Dichromate method or chloride ion calibration method	mg/L
Turbidity	TUR	Hach turbidity tester	NTU
Orthophosphate	PO_4^{3-}	Molybdenum-antimony anti-spectrophotometric method	mg/L
Ammonium nitrogen	NH_4^+	Nessler's reagent spectrophotometric method	mg/L
Nitrate	NO_3^-	Gas phase molecular Absorption spectrum method	mg/L
Nitrite	NO_2^-	Gas phase molecular Absorption spectrum method	mg/L
Dissolved inorganic nitrogen	DIN	$NH_4^+ + NO_3^- + NO_2^-$	mg/L

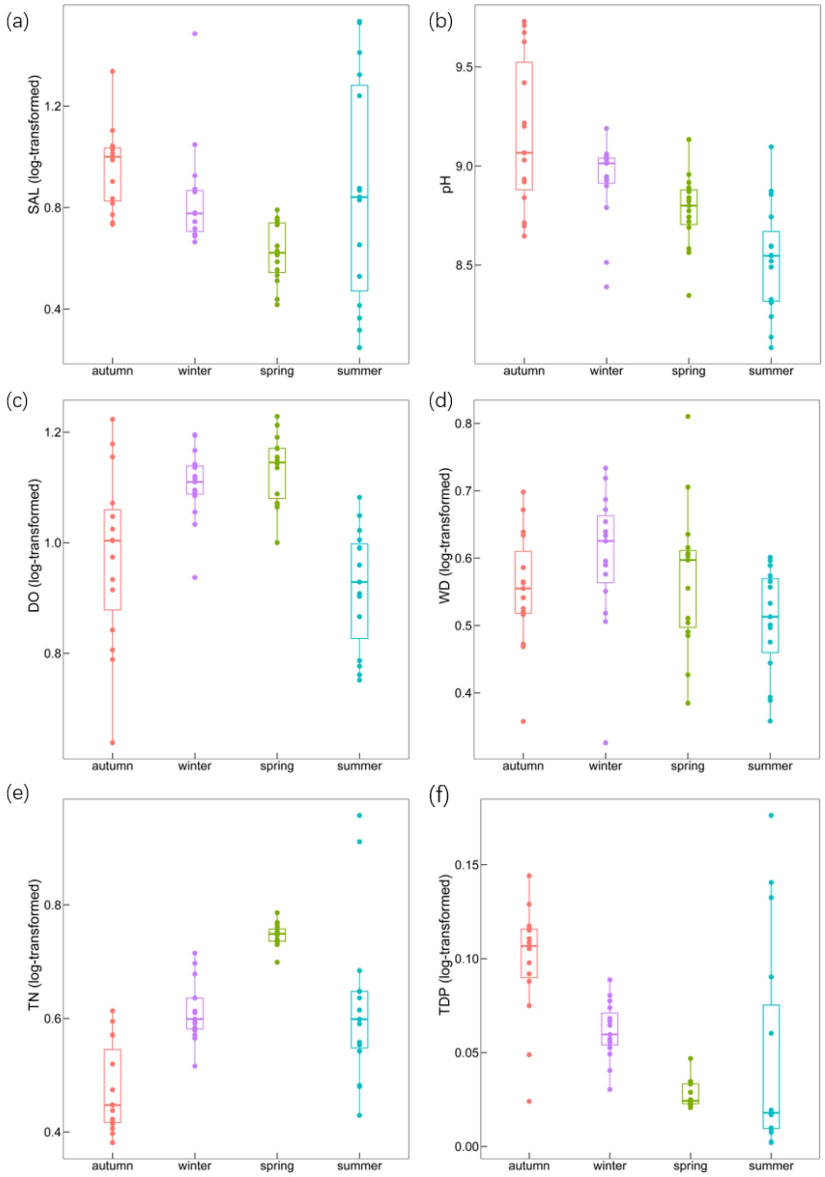

Figure S2. Seasonal variations of important environmental factors (All data has been log-transformed except pH). (a) Seasonal variations of SAL; (b) Seasonal variations of pH; (c) Seasonal variations of TN; (d) Seasonal variations of TDP; (e) Seasonal variations of DO; (f) Seasonal variations of WD; (g) Seasonal variations of WT. In the figure, the thickness of each box represents the average value, the maximum and minimum values above and below the vertical line, respectively, and the points away from the vertical line are outliers.

Table S2. Environmental factors in Duliujian River. Max = maximum values, Min = minimum values, SD = standard deviation. [1].

	On the annual scale			
**Environmental factors**	**Max**	**Min**	**Mean**	**SD**
pH	9.73	8.08	8.85	0.36
Salinity (ppt)	33.20	0.77	7.45	7.33
Total nitrogen (mg/L)	8.07	1.41	3.30	1.33
Total phosphorus (mg/L)	0.77	0.07	0.37	0.12
Total dissolved nitrogen (mg/L)	7.03	0.92	2.10	1.13
Total dissolved phosphorus (mg/L)	0.50	0.00	0.15	0.12
Orthophosphate (mg/L)	0.46	0.00	0.11	0.11

Dissolved inorganic nitrogen (mg/L)	2.37	0.11	0.83	0.65
Water temperature (℃)	29.27	1.73	17.77	10.30
Dissolved oxygen (mg/L)	15.92	3.35	10.27	3.16
Oxidation-reduction potential (mv)	221.93	70.47	132.77	32.09
Turbidity (NTU)	77.28	7.85	24.79	15.56
Water transparency (m)	0.99	0.27	0.49	0.13
Water depth (m)	5.46	1.12	2.70	0.84
Chemical oxygen demand (mg/L)	13.83	7.08	10.00	1.64
Ammonianitrogen (mg/L)	1.31	0.11	0.35	0.26
Nitrate nitrogen (mg/L)	1.68	0.00	0.42	0.46
Nitrite nitrogen(mg/L)	0.21	0.00	0.06	0.07

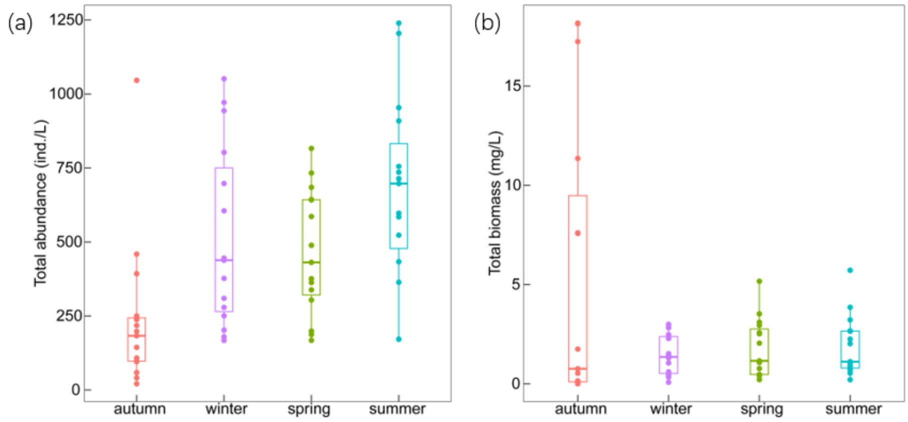

Figure S3. Seasonal variations of zooplankton abundance and biomass. (a) Seasonal variations of total abundance; (b) Seasonal variations of total biomass.

Table S3. Zooplankton species name and corresponding code.

Taxonomy	Species name	coding
Protozoa	*Tintinnopsis sinensis*	S1
	Stentor amethystinus	S2
	Vorticella lutea	S3
	Didinium balbianii	S4
	Pseudoprorodon armatus	S5
	Vorticella campanula	S6
Rotifers	*Epiphanes senla*	S7
	Keratella valga	S8
	Keratella cochlearis	S9
	Brachionus angularis	S10
	Brachionus calyciflorus Pallas	S11
	Brachionus urceus	S12
	Asplanchna brightwel	S13
	Filinia longisela	S14
Copepods	nauplius	S15
	copepodid larva	S16
	Calanus sinicus	S17
	Sinodiaptomus sarsi	S18
	Paracyclopina nana	S19
	Eucylops serrulatus	S20
	Ectocyclops phaleratus	S21

	Thermocyclops taihokuensis	S22
Cladocera	*Bosmina coregoni*	S23
	Bosmina longirostris	S24
	Diaphanosoma leuchtenbergianum	S25

Table S4. Spearman correlation coefficients of biomass of Protozoa and biomass of Bacillariophyta and Cryptophyta. *** means $p < 0.001$, ** means $p < 0.01$, *means $p < 0.05$.

	**Protozoa**	**Bacillariophyta**	**Cryptophyta**
Protozoa	1	0.38**	0.35**
Bacillariophyta	0.38**	1	0.70**
Cryptophyta	0.35**	0.70**	1

References

1. Sun, X.; Zhang, H.; Wang, Z.; Huang, T.; Huang, H. Phytoplankton Community Response to Environmental Factors along a Salinity Gradient in a Seagoing River, Tianjin, China. *Microorganisms* **2022**, *11*, 75, doi:10.3390/microorganisms11010075.
2. Ministry of Environmental Protection of the People's Republic of China. *In Determination Methods for Examination of Water and Wastewater, 4th Ed*; China Environmental Science Press: Beijing, China, 2002.
